# Alexithymia and the Evaluation of Emotionally Valenced Scenes

**DOI:** 10.3389/fpsyg.2020.01820

**Published:** 2020-07-24

**Authors:** Sarah N. Rigby, Lorna S. Jakobson, Pauline M. Pearson, Brenda M. Stoesz

**Affiliations:** ^1^ Department of Psychology, University of Manitoba, Winnipeg, MB, Canada; ^2^ Department of Psychology, University of Winnipeg, Winnipeg, MB, Canada; ^3^ Centre for the Advancement of Teaching and Learning, University of Manitoba, Winnipeg, MB, Canada

**Keywords:** alexithymia, emotion, implied motion, scene perception, sensory processing sensitivity

## Abstract

Alexithymia is a personality trait characterized by difficulties identifying and describing feelings (DIF and DDF) and an externally oriented thinking (EOT) style. The primary aim of the present study was to investigate links between alexithymia and the evaluation of emotional scenes. We also investigated whether viewers’ evaluations of emotional scenes were better predicted by specific alexithymic traits or by individual differences in sensory processing sensitivity (SPS). Participants (*N* = 106) completed measures of alexithymia and SPS along with a task requiring speeded judgments of the pleasantness of 120 moderately arousing scenes. We did not replicate laterality effects previously described with the scene perception task. Compared to those with weak alexithymic traits, individuals with moderate-to-strong alexithymic traits were less likely to classify positively valenced scenes as pleasant and were less likely to classify scenes with (vs. without) implied motion (IM) in a way that was consistent with normative scene valence ratings. In addition, regression analyses confirmed that reporting strong EOT and a tendency to be easily overwhelmed by busy sensory environments negatively predicted classification accuracy for positive scenes, and that both DDF and EOT negatively predicted classification accuracy for scenes depicting IM. These findings highlight the importance of accounting for stimulus characteristics and individual differences in specific traits associated with alexithymia and SPS when investigating the processing of emotional stimuli. Learning more about the links between these individual difference variables may have significant clinical implications, given that alexithymia is an important, transdiagnostic risk factor for a wide range of psychopathologies.

## Introduction

Alexithymia is a personality trait characterized by deficits in understanding one’s emotions (Nemiah et al., 1976). Associated problems with emotion perception have also been described, particularly in the area of facial expression recognition (Parker et al., 2005; Vermeulen et al., 2006; Grynberg et al., 2012; Jongen et al., 2014). How this trait influences emotional scene perception is less well understood. Extending our knowledge in this area is important, given that (a) integrating various types of cues (e.g., facial expressions, body postures, surrounding objects, and context) is essential for optimal emotion understanding in everyday life and (b) individual differences are important to consider (rather than control for) when researching visual scene perception (de Haas et al., 2019). The main goal of the current study was to investigate relationships between alexithymic traits and the evaluation of emotional scenes. Additionally, we aimed to elucidate whether individuals with strong alexithymic traits showed signs of atypical hemispheric contributions to emotional scene perception. Finally, we investigated whether viewers’ evaluations of emotional scenes were better predicted by specific alexithymic traits or by individual differences in sensory sensitivity.

To provide context for the present study, we begin by summarizing some of the relevant literature on scene perception. Scenes depict real-world environments seen from a particular viewpoint. They represent our “natural visual input” (Groen et al., 2017, p. 1), encompassing objects and spatial features appearing in both foveal and peripheral vision. According to interaction-based views of scene understanding, observers place themselves “within” the scenes they view, extracting information not only about low-level features (e.g., edges, spatial frequencies) but also about high-level characteristics, including the affordances and contextual associations between specific features (see Malcolm et al., 2016). Bottom-up and top-down processes interact during scene processing, with the relative weight given to each process changing as a function of the viewer’s behavioral goal (e.g., recognition, navigation; Groen et al., 2017), and how strongly elements in a scene resonate with the viewer on an emotional level (Kuniecki et al., 2017). The nonhierarchical nature of emotional scene processing is illustrated by work showing enhanced activity in, and *bidirectional connectivity between*, broad regions of visual cortex, the frontoparietal network, and anterior corticolimbic structures during viewing of emotionally arousing (vs. neutral) scenes (Frank et al., 2019). Different parts of this interactive network play key roles in determining stimulus value, updating reward contingencies, and modulating attention to emotional stimuli (Frank and Sabatinelli, 2019).

The idea that emotionally arousing scenes are processed differently from neutral scenes gains support from a wealth of functional imaging studies showing differential patterns of brain activity associated with viewing emotional and neutral scenes (see Sabatinelli et al., 2011). But there are also differences in how we process and respond to positively and negatively valenced scenes. At a neural level, one sees enhanced neural responses to pleasant (vs. unpleasant) scenes across regions that include sensory and prefrontal areas (Junghofer et al., 2017; Frank and Sabatinelli, 2019). At a behavioral level, viewers often require less time to categorize pleasant scenes and more time to process unpleasant scenes relative to scenes with neutral content, particularly when images are high in intensity (Ihssen and Keil, 2013). These findings suggest that pleasantness acts as a general “safety signal” (motivating approach), whereas arousing, unpleasant stimuli signal the need to respond carefully to avoid making errors that might place one in danger. Whether an approach, an avoidance, or a freezing response is most appropriate will depend on the particular situation (Kuhbandner et al., 2016; Roelofs, 2017). In this respect, the processing of negatively valenced scenes may be considered more complex than the processing of positively valenced scenes.

If negative scenes are more complex than positive ones, their processing may require greater interhemispheric interaction (e.g., Weissman and Banich, 2000; Shobe, 2014). This idea was tested by Hughes and Rutherford (2013). They developed a paradigm to investigate hemispheric contributions to the processing of emotional stimuli, and used it to test viewers’ evaluations of images obtained from the International Affective Picture System (IAPS; Lang et al., 2008). Unlike the divided visual field technique, Hughes and Rutherford’s task requires viewers to make judgments regarding stimuli presented *centrally* for relatively long durations (2.5 s). On some trials, a flashing square is presented in the left or right periphery early during stimulus presentation to draw attention and thereby “distract” the contralateral hemisphere—ostensibly shifting processing more heavily toward the ipsilateral hemisphere. Hughes and Rutherford contend that comparing participants’ performance during left and right distractor trials provides an index of laterality, and that adding a no distractor condition allows one to explore the possible advantages of hemispheric interaction. The results of their study on asymmetry in the processing of emotional scenes were mostly in line with the valence hypothesis (a right hemisphere advantage for negative and a left hemisphere advantage for positive scenes). Hughes and Rutherford also found, however, that processing negatively valenced scenes benefitted more from hemispheric interaction than processing positive scenes, supporting the idea that negative scenes are more complex.

Other stimulus characteristics also undoubtedly increase scene complexity. In the current investigation, we were interested in the extent to which the presence or absence of implied motion (IM) influenced how images of different kinds of scenes were evaluated. Movement can be suggested visually in a static image in a variety of ways. The idea that the presence of IM makes the processing of an image more complex is supported by the observation that movement information captured in “frozen action” photos (e.g., a person diving into water or a rocket taking off) gets incorporated into our representations of scenes, leading to memory biases (Freyd and Finke, 1984). It has also been shown that viewing IM images recruits areas of the brain important for processing real motion (Kourtzi and Kanwisher, 2000) and emotional information (Kolesar et al., 2017), and that IM can modulate how emotionally valenced images are processed. In one study, images of body postures that implied either action *or* emotion were found to produce larger motor evoked potentials (MEPs) than images of bodies at rest or in emotionally neutral postures (Borgomaneri et al., 2012). In a subsequent investigation, Borgomaneri et al. (2015) found evidence that people oriented to emotional IM early in processing and engaged in simulation of both emotional and neutral actions later in processing. If IM does increase scene complexity, one might predict that the processing of scenes containing IM (like those with negative valence) would benefit from hemispheric interaction. Regardless, it seems clear that both valence and IM impact scene processing, and that it is important to consider the content of scenes carefully if one hopes to disentangle the effects of these variables on scene processing.

There is increasing interest in exploring factors that may underlie individual differences in scene processing (e.g., Hayes and Henderson, 2017, 2018; de Haas et al., 2019). The overarching goal of the present research was to add to this literature by exploring how individuals reporting different levels of alexithymia process and evaluate different kinds of emotional scenes. Alexithymia is a trait represented in the general population (Salminen et al., 1999) and characterized by difficulties identifying and describing feelings (DIF and DDF, respectively), externally oriented thinking (EOT), and an impoverished fantasy life (Nemiah et al., 1976). Some suggest that interoceptive impairments underlie some of these features (e.g., Murphy et al., 2018), but alexithymia is also linked to atypical processing of environmental stimuli. For example, Liss et al. (2008) noted overlap between alexithymia and particular aspects of sensory processing sensitivity (SPS). Individuals scoring high in SPS are said to have rich inner lives and to engage in deep/complex processing; they also exhibit heightened emotional reactivity to and are easily overwhelmed by both positively and negatively valenced environmental stimuli, and approach novel situations cautiously (Aron et al., 2012; Lionetti et al., 2018). In their work, Liss et al. found that EOT was negatively correlated with the tendency to be emotionally “moved” by music and the arts (aesthetic sensitivity), and that DIF and DDF were positively correlated with heightened emotional reactivity to environmental stimuli.

We propose that individuals with alexithymia process, integrate, and respond to sensory information in unusual ways, and that this impacts how scenes are embodied and evaluated. Consistent with this idea, several brain regions that show greater activation during viewing of emotional (vs. neutral) scenes (see Sabatinelli et al., 2011) have also been implicated in alexithymia (see Bermond et al., 2006). Impaired interhemispheric transfer has also been described in alexithymia (see Bermond et al., 2006), and this might be significant given the proposed role of interhemispheric communication in late stages of scene processing (see Groen et al., 2017). Past work suggests that, compared to lexithymic individuals, those scoring high on alexithymia spend less time looking at faces within scenes (Bird et al., 2011). This atypical attentional guidance may explain why alexithymia is associated with a reduced ability to determine the congruency between facial expressions and scene content (Lane et al., 1996, 2000), and with a failure to respond quickly (as lexithymic individuals do) to subtle changes in facial expressions that are consistent (vs. inconsistent) with changes in scene valence (Yamashita et al., 2016).

Early reports indicated that individuals with alexithymia respond differently than controls to cues in scenes that signal arousal, but not to those that signal valence (Roedema and Simons, 1999; see also Wehmer et al., 1995). However, in subsequent work utilizing a much larger stimulus set, Koven (2014) found that individuals with alexithymia under-rated the valence of positive/appetitive scenes (but see Heinzel et al., 2010). Others have reported that specific alexithymic traits differentially predict viewers’ physiological and behavioral responses to negatively valenced scenes. For example, Berenbaum (1996) found that high DIF scores predicted stronger *preferences* for negatively valenced (vs. happy) films. In addition, high EOT scores have been found to predict shorter dwell times on depression-related (but not anxious, neutral, or positive) images (Wiebe et al., 2017) and reduced physiological reactivity during viewing of sad films (Davydov et al., 2013). Aaron et al. (2018) suggested that EOT is associated with reduced *awareness* of one’s emotional states and with a reduced experience of mixed emotional reactions to negatively valenced films. They proposed that EOT may be a stable trait that predicts one’s aptitude for emotion processing, whereas DIF and DDF may be more state-like and predict one’s tendency to engage in emotion processing in real-life. Aaron et al. highlight the need for more research aimed at improving our understanding of factors underlying these different features of alexithymia.

In the natural world, we often interpret and react to emotional information conveyed, in part, by movement. Several findings suggest that introduction of movement cues might increase the processing challenge for people with stronger alexithymic traits. For example, alexithymia is associated with an attentional bias toward perceptual motion cues at the expense of socially relevant gaze cues (Wei et al., 2019), and with reduced confidence (but not accuracy) in valence judgments for emotions conveyed in whole-body point-light displays (Lorey et al., 2012). In other work, Borhani et al. (2016) showed atypical fear-related modulation of early perceptual processing of IM in those scoring high on alexithymia. Finally, high Toronto Alexithymia Scale (TAS-20) Total and EOT scores have also been shown to predict reduced incorporation of IM into memory representations for facial expressions (Senior et al., 2020). To our knowledge, relationships between alexithymia and the processing of IM in emotional *scenes* have not been reported, but it would be of interest to explore this given that the neural substrates of scene processing differ from those underlying face and body processing (e.g., Pitcher et al., 2019), and that (as discussed above) IM is known to impact scene processing.

How, or if, hemispheric contributions to emotion scene perception are altered in those with alexithymia is unclear. Past work suggests that alexithymia is associated with increased right hemisphere malfunction, inhibition, or impairment (Kano et al., 2003; Aftanas and Varlamov, 2004; Bermond et al., 2006; Ricciardi et al., 2015); left hemisphere hyperactivation or biases (Bermond et al., 2006; Karlsson et al., 2008); and deficits in interhemispheric transfer of emotion information (Parker et al., 1999; Liemburg et al., 2012; Shobe, 2014). Any (or all) of these problems could theoretically disrupt cue integration during the processing of complex emotional stimuli, such as those depicting IM.

In the present study, we employed the paradigm developed by Hughes and Rutherford (2013) to study relationships between alexithymia and the processing of static emotional scenes that do or do not depict IM. We expected that positive images would be easier to classify than negative images, overall, replicating findings of positivity biases in emotion perception (Zhao et al., 2017). Moreover, if negative valence and IM increase scene complexity, we reasoned that participants would generally be slower and less accurate when classifying scenes with these attributes, compared to ones that were positively valenced and did not imply motion. Importantly, however, we also predicted that people reporting high (vs. low) levels of alexithymia would find it more challenging to categorize scenes (particularly those depicting IM) quickly and accurately, and that they might show unusual laterality effects. Relationships between specific alexithymic traits and task performance were explored using a regression-based approach. We accounted for individual variation in traits associated with SPS in these analyses to determine whether performance with specific types of stimuli was better predicted by particular alexithymic traits or by traits associated with SPS.

## Materials and Methods

### Participants

We recruited participants through the Introduction to Psychology participant pool at the University of Manitoba; each received credit toward a course requirement. One male participant did not complete the alexithymia measure, so his data were excluded from all analyses. This left a final sample of *N* = 106 (64 women and 42 men, aged 18–31 years; *M* = 21, *SD* = 2.8), which was large enough to allow us to detect (at the 0.05 probability level) a medium effect size in our planned regression analyses at least 80% of time.

Participation was restricted to individuals who self-reported being right-handed and having normal or corrected-to-normal vision. Handedness was later confirmed through administration of a questionnaire (see below). All participants reported having normal developmental histories, and no previous diagnosis of a neurological disorder or significant head injury.

### Procedure

The Psychology/Sociology Human Research Ethics Board at the University of Manitoba approved the testing protocol. All participants gave informed consent to take part and were tested individually in a quiet, dimly lit room. Participants completed the Positive and Negative Affect Schedule—short form (PANAS; Thompson, 2007), the Edinburgh Handedness Inventory (Oldfield, 1971), and the emotional scenes task, in that order. The experimental task was explained verbally to each participant before they began the task and participants could ask questions during this time. Following the emotional scenes task, participants completed a demographics questionnaire, the TAS-20 (Bagby et al., 1994) and the Highly Sensitive Person Scale (HSPS; Aron and Aron, 1997). The order in which the last two questionnaires were completed was counterbalanced across participants.

### Materials

#### Positive and Negative Affect Schedule – Short Form

We administered the PANAS prior to the experimental task to allow us to rule out the possibility that low mood confounded the results. This 10-item self-report questionnaire was derived from the original PANAS (Watson et al., 1998). Five items assess positive affect (i.e., alert, inspired, determined, attentive, and active), and five items assess negative affect (i.e., upset, hostile, ashamed, nervous, and afraid). Participants indicated the extent to which they felt each of the emotions in the present moment on a five-point Likert scale, ranging from 1 = *Very slightly or not at all* to 5 = *Extremely*. The PANAS shows adequate reliability and validity (Thompson, 2007).

#### Edinburgh Handedness Inventory

The Edinburgh Handedness Inventory (Oldfield, 1971) is a 12-item questionnaire used to assess hand dominance. In completing the inventory, participants indicated whether they preferred to use their right or left hand for a variety of unimanual activities; if they would never use their other hand unless they were absolutely forced to; or whether they were indifferent to which hand was used. A laterality quotient was computed for each participant, with positive scores indicating right-handedness and larger absolute scores indicating stronger handedness.

#### Toronto Alexithymia Scale – 20

The TAS-20 (Bagby et al., 1994) is comprised of 20 items that contribute to three subscales assessing core features of alexithymia: DIF (seven items); DDF (five items), and EOT (eight items). For each item, participants responded using the five-point Likert scale ranging from 1 = *Strongly disagree* to 5 = *Strongly agree*. Total scores can range from 20 to 100.

#### Highly Sensitive Person Scale

The HSPS measures aspects of SPS (Aron and Aron, 1997). Participants responded to each of the 27 items using a seven-point Likert scale ranging from 1 = *Not at all* to 7 = *Extremely*. A mean score is computed to obtain a total score out of seven. Based on confirmatory factor analyses, Smolewska et al. (2006) proposed that the HSPS measures three specific aspects of SPS: ease of excitation (EOE), low sensory threshold (LST), and aesthetic sensitivity (AES). The EOE subscale (12 items) taps into how easily overwhelmed one is by internal and external stimuli and by multi-tasking demands. The LST subscale (six items) addresses the extent to which one feels uncomfortable with certain kinds of sensory experiences, and how strongly one seeks to avoid them. The AES subscale (seven items) assesses the extent to which one feels “moved” by the arts. Subscale scores are obtained by averaging responses on relevant items.

#### Emotional Scenes Task

As in Hughes and Rutherford’s (2013) protocol, when completing the emotional scenes task participants made speeded judgments of whether photographs obtained from the IAPS (Lang et al., 2008) were pleasant or unpleasant. Normative arousal and valence ratings have been compiled for this picture set using scales that range from 0 = *Low arousal/Unpleasant* to 9 = *High arousal/Pleasant*. Images selected for this investigation (*N* = 120) had moderate arousal ratings (means ranging from 4 to 6). Half were negatively valenced (mean valence ratings 2–3.99; e.g., a car crash), and half were positively valenced (mean valence ratings 6.01–8; e.g., smiling children), and within each of these sets half of the images depicted IM (e.g., a tornado) and half did not (e.g., a mountain). In sets of images with a given valence, images with vs. without IM had comparable mean valence ratings (paired *t*-tests: positive images, *p* = 0.69; negative images, *p* = 0.71). Finally, images across all four sets had comparable subjective arousal ratings (*p* ≥ 0.11) and were balanced with regard to content, with approximately two-thirds of the images of each type depicting humans or non-human animals, and the remainder depicting scenes from nature or inanimate objects. In the majority of cases, the most salient content fell near the center of the image. In a few cases, the most salient content was displaced toward the left (four negative and three positive images) or the right (four negative and five positive images); one of the positive images in the latter set was mirror-reversed, so that an equal number of images of each valence had a focal element that was displaced to the left or the right.

The procedures followed were closely modeled on the paradigm outlined by Hughes and Rutherford (2013). The task was created using E-Prime 2.0 (Psychology Software Tools, 2012) and was presented to participants on a PC computer. Each participant rested his/her chin on a chin rest to ensure that head position was aligned with the center of the screen, at a viewing distance of approximately 57 cm. Each trial began with presentation of a central fixation cross for 500 ms, followed by a central stimulus image for 2.5 s. Stimulus images subtended a visual angle of 2.9° in height and 3.9° in width. A small white square (0.41° in height and width) served as the distractor. The distractor appeared 7.4° to the right of center on one-third of the trials of each type; it appeared 7.4° to the left of center on one-third of the trials; and no distractor was presented on the remaining trials. When present, the appearance of the distractor coincided with presentation of the stimulus image, and the distractor then blinked on-and-off at 50 ms intervals for 300 ms.

On each trial, participants were asked to classify the image presented as pleasant or unpleasant as quickly and accurately as possible using the keyboard. *Pleasant* was equated with making the participant feel “happy, pleased, satisfied, contended, or hopeful,” whereas *unpleasant* was equated with making the participant feel “unhappy, annoyed, unsatisfied, melancholic, despaired, or bored” (as per Hughes and Rutherford, 2013, p. 170). To reduce stimulus response compatibility effects that might be associated with presentation of the distractors, participants made bimanual responses; half of the participants pressed the “f” and “j” keys simultaneously with their index fingers for pleasant images and pressed the “d” and “k” keys simultaneously with their middle fingers for unpleasant images; key assignments were reversed for the remaining participants. Trials ended when a response was made or 2.5 s after stimulus onset (whichever came first), and the next trial began after the participant pressed the space bar. Participants completed 18 practice trials (three positive and three negative trials in each distractor condition), featuring scenes not included in the experimental set. They then completed one experimental block consisting of 120 trials. Stimulus order within the experimental block was randomized for each participant. Accuracy and response time (RT) data were collected for each trial.

## Results

We used analysis of variance (ANOVA) and hierarchical multiple regressions to explore relationships between our study variables. We completed all analyses using SPSS 25 (Armonk, NY: IBM Corp.) and adopted an alpha level of 0.05 for tests of significance. Scores on the Edinburgh Handedness Inventory confirmed that all participants were right-handed (*M* = 79.4, *SD* = 23, *Range* = 13–100). Strength of handedness was unrelated to task performance and was not considered further.

### Effects of Alexithymia and Stimulus Type on Task Performance

As a first step, mean RT on correct trials and mean accuracy in the various conditions of the emotional scenes task were submitted to separate 3 (Alexithymia Group: Low, Moderate, High) × 2 (Valence: Positive, Negative) × 3 (Distractor Location: Right, Left, None) × 2 (IM: Present, Absent) ANOVAs, with repeated measures on the last three factors. Significant main effects and interactions were followed up with LSD tests and tests of simple main effects, respectively.

TAS-20 Total scores were normally distributed in our sample, *W*(106) = 0.99, *p* = 0.56. Following Bagby et al. (2020) recommendation that previously established cut-scores not be used in research on alexithymia, we used tertiles of the distribution of TAS-20 Total scores to classify participants as low, moderate, or high in alexithymic traits. This allowed for the creation of three groups of equal size. The mean Total scores of the Low Alexithymia (LA), Moderate Alexithymia (MA), and High Alexithymia (HA) groups were 37.5 (*SD* = 4.5), 47.6 (*SD* = 2.4) and 59.3 (*SD* = 5.3), respectively. The three groups had comparable sex distributions, *χ* ^2^(2) = 0.82, *p* = 0.66, and self-reported negative affect, *F*(2, 103) = 0.66, *p* = 0.52. As such, neither sex nor PANAS-negative scores were included in the ANOVAs.

#### Mean RT on Correct Trials

Before analyzing the RT data, distributions were winsorized by replacing the 0.2% of mean RTs that were >3.25 SD above the overall mean with the next slowest mean RT in the corresponding condition that was not an outlier. The resulting distributions had acceptable skewness and kurtosis.

Based on past research (Hughes and Rutherford, 2013), we predicted a significant distractor location × valence interaction, but this was not supported. Indeed, there were no significant interactions involving distractor location (0.17 ≤ *p* ≤ 0.87). There was, however, a significant main effect of distractor location, *F*(2, 206) = 64.12, *p* < 0.001, *η_p_* ^2^ = 0.38, with participants responding more slowly in the no distractor than in the right or left distractor conditions (*p* < 0.001), which themselves did not differ (*p* > 0.99 for both contrasts).

We found main effects of valence, *F*(1, 103) = 5.11, *p* = 0.026, *η_p_* ^2^ = 0.047, and IM, *F*(1, 103) = 6.67, *p* = 0.011, *η_p_* ^2^ = 0.061. Participants responded more quickly to positive images and to images that did not depict IM (*p* < 0.001 for both contrasts). There were no effects or interactions involving alexithymia group in the analysis of the RT data.

#### Accuracy

Accuracy was defined as classifying a scene in a way that was consistent with its normative valence rating (i.e., with rating a positive scene as “pleasant” and a negative scene as “unpleasant”). Before analyzing the accuracy data, distributions were winsorized by replacing the 1.1% of accuracy scores that were >3.25 SD below the mean with the next lowest accuracy score in the corresponding condition that was not an outlier. The resulting distributions exhibited acceptable skewness and kurtosis.

**Figure 1:**
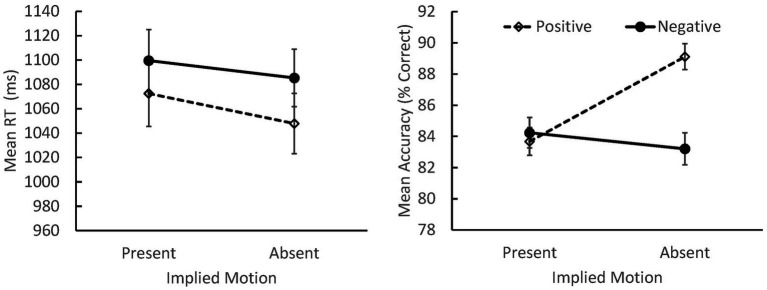
Mean response times (RTs; **left panel**) and accuracy **(right panel)** for judgments of positively and negatively valenced scenes that did or did not depict IM (SEs indicated). Overall, participants responded more quickly to positive than negative images, and to images that did not depict IM. Accuracy was highest when classifying positive scenes without IM.

There were no main effects or interactions involving distractor location (0.24 ≤ *p* ≤ 0.93). As with the RT data, we observed main effects of valence, *F*(1, 103) = 4.05, *p* = 0.047, *η_p_* ^2^ = 0.038, and IM, *F*(1, 103) = 18.54, *p* < 0.001, *η_p_* ^2^ = 0.153, along with a significant valence × IM interaction, *F*(1, 103) = 33.83, *p* < 0.001, *η_p_* ^2^ = 0.247 (see Figure 1). Overall, viewers were more accurate when classifying positive images without IM than in any other condition (*p* < 0.001 for all contrasts) suggesting that these images were the easiest to evaluate, overall.

**Figure 2:**
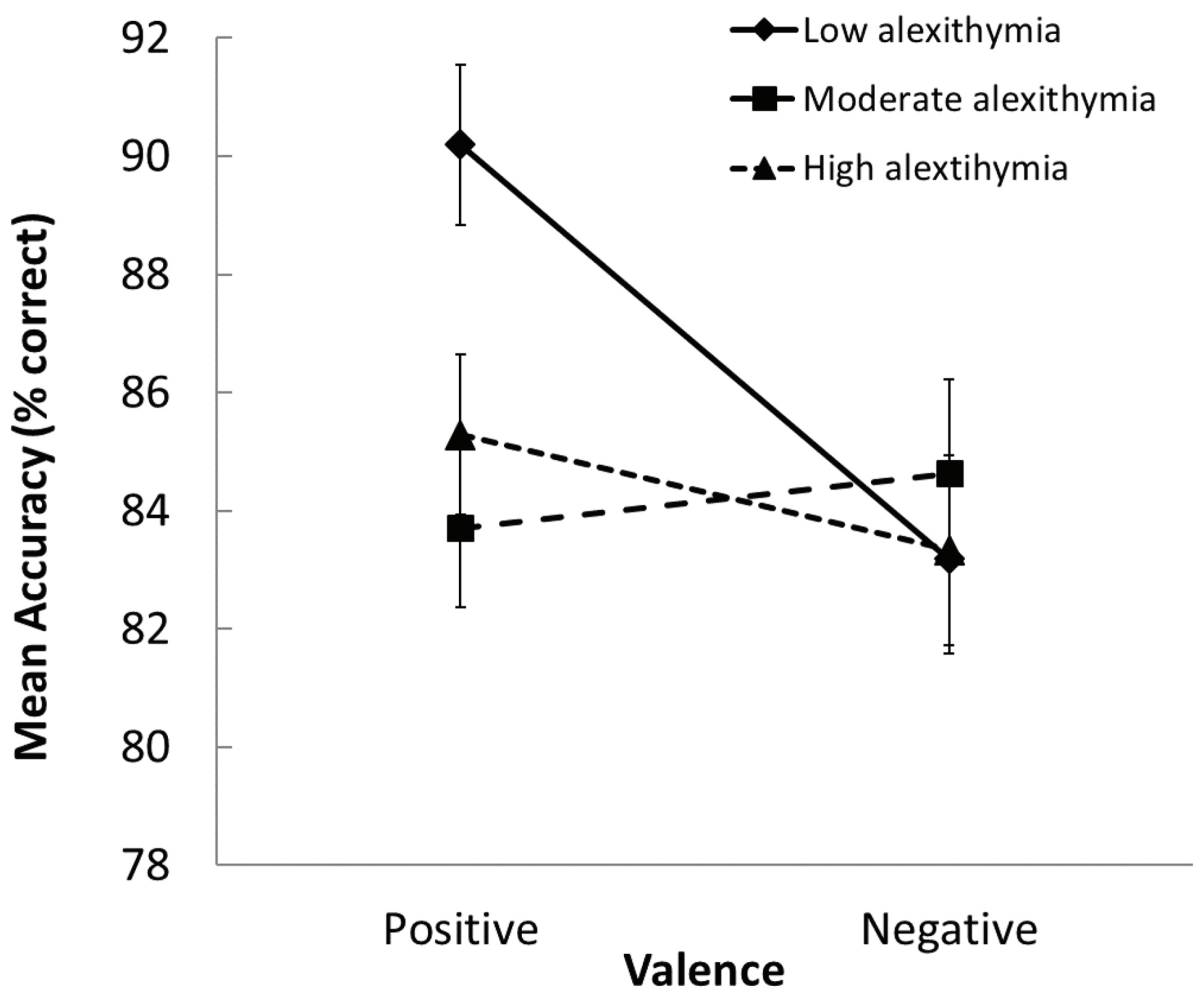
Group differences in how accurately positively and negatively valenced scenes were evaluated (SEs indicated). Participants with moderate-to-high alexithymic traits were less accurate at judging the valence of positive scenes than those with low alexithymic traits.

We also observed a significant group × valence interaction in the accuracy data, *F*(2, 103) = 3.04, *p* = 0.05, *η_p_* ^2^ = 0.056 (see Figure 2). The LA group was more accurate than the MA and HA groups when classifying positive scenes, and only the LA group classified positive scenes more accurately than negative ones (*p* ≤ 0.003 for both contrasts). This pattern of results suggests the presence of an underlying “positivity bias” in the LA group. This was not evident in the MA and HA groups; thus, they exhibited similar accuracy in the classification of both types of scenes.

**Figure 3:**
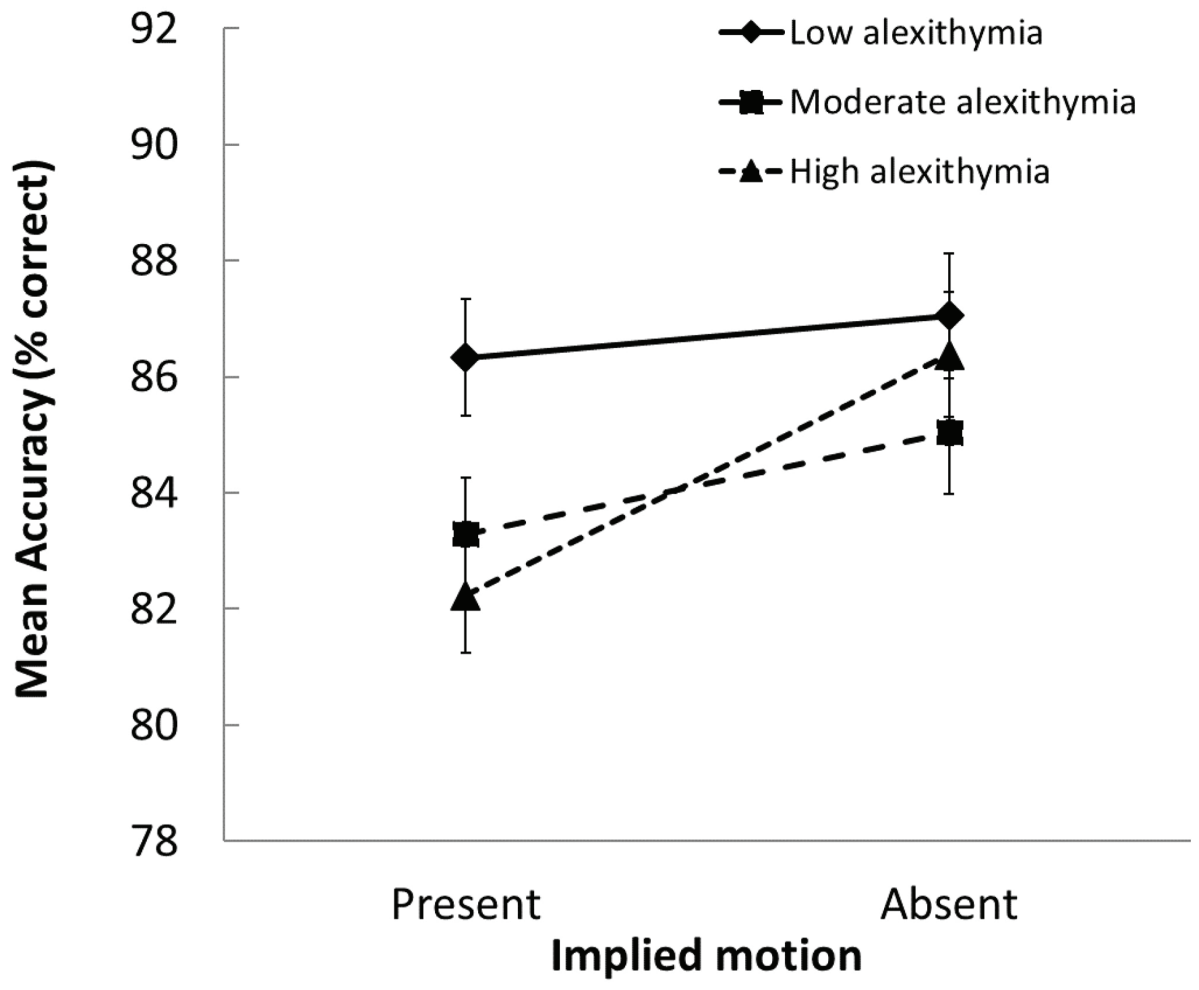
Group differences in how accurately scenes with vs. without IM were evaluated (SEs indicated). Participants with moderate and high alexithymic traits were less accurate at judging the valence of scenes that depicted IM than those with low alexithymic traits.

Follow-up tests on the significant group × IM interaction, *F*(2, 103) = 3.89, *p* = 0.024, *η_p_* ^2^ = 0.07, showed that the LA group classified scenes with IM more accurately than the MA and HA groups (*p* ≤ 0.032 for both contrasts). In addition, whereas the LA group was equally successful at classifying scenes with and without IM, the MA and HA groups were less likely to classify scenes with (vs. without) IM in a way that was consistent with normative scene valence ratings (*p* ≤ 0.048 for both contrasts; see Figure 3).

### Variance Accounted for by Individual Traits Associated With Alexithymia and SPS

TAS-20 Total scores were significantly correlated with HSPS Total scores, and significant correlations were observed between specific subscales of the two measures (see Table 1). In addition to examining these correlations, we also looked at differences in the distributions of HSPS scores in the three groups, using the 30th and 70th percentiles to group individuals according to low, moderate, and high levels of SPS (as per Lionetti et al., 2018). The distributions of these subtypes were different across the three alexithymia groups, *χ* ^2^(4) = 12.12, *p* = 0.016, with the proportion of individuals scoring high on SPS increasing from LA (14.3%), to MA (33.3%), to HA (48.6%).

**Table 1:** Correlations between measures of alexithymia and sensory processing sensitivity (SPS).

		TAS-20				HSPS		
		Total	DDF	DIF	EOT	Total	EOE	AES
TAS-20	Total	--						
	DDF	0.75^**^	--					
	DIF	0.78^**^	0.46^**^	--				
	EOT	0.57^**^	0.18	0.14	--			
HSPS	Total	0.26^**^	0.23^*^	0.42^**^	−0.13	--		
	EOE	0.33^**^	0.29^**^	0.46^**^	−0.06	0.84^**^	--	
	AES	−0.06	−0.02	0.12	−0.27^**^	0.53^**^	0.25^**^	--
	LST	0.17	0.11	0.25^*^	−0.02	0.75^**^	0.46^**^	0.13

TAS-20, Toronto Alexithymia Scale; DIF, difficulty identifying feelings; DDF, difficulty describing feelings; EOT, externally oriented thinking subscales; HSPS, Highly Sensitive Person Scale; EOE, ease of excitation; AES, aesthetic sensitivity; LST, low sensory threshold subscales.

* Correlation is significant at the 0.05 level (2-tailed);

** Correlation is significant at the 0.01 level (2-tailed).

Given the high rates of SPS in individuals with alexithymia, we considered the possibility that the observed links between task performance and alexithymia might, in fact, be related to individual differences in traits related to SPS. To test this, we ran four separate multiple regressions predicting the number of correctly classified positive, negative, IM, and no-IM scenes. As distractor location had no impact on classification accuracy (see above), responses to trials in different distractor conditions were averaged when computing output variables. We used the forced entry method, entering sex, PANAS-negative scores (to account for negative affect), the three TAS-20 subscale scores (EOT, DIF, and DDF), and the three HSPS subscores (EOE, LST, and AES) as predictors in each regression. Before proceeding, we confirmed that multicollinearity was not an issue (variance inflation factor ≤1.72 in all cases).

Significant models were only observed in the two conditions in which group differences had been observed in the ANOVAs, namely in the classification of positive scenes and scenes depicting IM. The overall models in both analyses were significant (*R* ^2^ ≥ 0.18, *p* ≤ 0.01; *f* ^2^ ≥ 0.22). As can be seen in Table 2, both EOT and EOE were significant negative predictors of accuracy in classifying positive scenes. Thus, individuals reporting a stronger external focus and those reporting a tendency to become easily overwhelmed by busy sensory environments were less likely to classify positive scenes as pleasant compared to those scoring low on these traits. DDF and EOT scores significantly predicted accuracy in the classification of scenes depicting IM, with those scoring high on these scales being less likely to classify scenes with (vs. without) IM in a way that was consistent with normative scene valence ratings, compared to those scoring low. Sex was a significant predictor in both models, with males classifying both types of scenes in a way that was more consistent with normative ratings. This may relate to the fact that the scenes were classified on a coarse level (pleasant/unpleasant). Past research suggests that women show more variability than men in how they rate emotional scenes (e.g., Roedema and Simons, 1999). This might be because they make more fine-grained distinctions between their emotional responses (Barrett et al., 2000), which could impact their overall assessments.

**Table 2:** Linear models predicting accuracy in judgments of positive and implied motion (IM) scenes.

		*b*	*SE b*	*β*	*p*
Positive Scenes	(Constant)	68.343	4.717		0
	**Sex**	−**2.602**	**1.022**	−**0.253**	**0.013**
	PANAS Neg	0.031	0.196	0.015	0.873
	DIF	0.002	0.104	0.003	0.981
	DDF	−0.083	0.134	−0.065	0.534
	**EOT**	−**0.301**	**0.112**	−**0.257**	**0.009**
	**EOE**	−**1.430**	**0.687**	−**0.245**	**0.040**
	AES	0.046	0.601	0.008	0.939
	LST	0.166	0.387	0.044	0.669
Implied Motion Scenes	(Constant)	60.561	3.502		0
	**Sex**	−**1.757**	**0.759**	−**0.236**	**0.023**
	PANAS Neg	−0.065	0.145	−0.043	0.656
	DIF	−0.001	0.077	−0.001	0.992
	**DDF**	−**0.202**	**0.099**	−**0.216**	**0.045**
	**EOT**	−**0.215**	**0.083**	−**0.253**	**0.011**
	EOE	−0.034	0.510	−0.008	0.947
	AES	−0.024	0.446	−0.005	0.957
	LST	0.029	0.287	0.010	0.920

PANAS Neg, PANAS negative affect subscale; TAS-20, Toronto Alexithymia Scale; DIF, difficulty identifying feelings; DDF, difficulty describing feelings; EOT, externally oriented thinking subscales; HSPS, Highly Sensitive Person Scale; EOE, ease of excitation; AES, aesthetic sensitivity; LST, low sensory threshold subscales. Bolded values are significant at p < 0.05 or below.

## Discussion

This study was designed to investigate the impact of individual variations in alexithymia and stimulus characteristics on the evaluation of emotionally valenced scenes. We found that participants generally classified positive scenes without IM more quickly and accurately than other types of scenes. Accounting for alexithymia was important, however, as participants reporting moderate-to-high levels of alexithymia classified positively valenced scenes and scenes with IM less accurately than those reporting low levels of this trait. Regression analyses revealed that EOT scores drove the relationship between alexithymia and accuracy in the evaluation of positive scenes, but one aspect of SPS – being bothered by busy sensory environments (EOE) – also accounted for unique variance in how accurately these scenes were classified. Both EOT and DDF predicted accuracy in classification of IM scenes. We did not find evidence of the expected laterality effects in the emotional scenes task in the sample as a whole or in subgroups distinguished by different levels of alexithymia. These key findings are discussed below.

### Hemispheric Contributions to Task Performance

Contrary to an earlier report using the same paradigm (Hughes and Rutherford, 2013), we found that participants were faster to respond to images when a distractor was present (unilateral conditions) compared to when no distractor was present (bilateral condition). One possible explanation for this finding is that the emotional scenes task was relatively easy and could be completed well unilaterally. (Note that engagement of both hemispheres during cognitively simple tasks has been shown to attenuate performance; Weissman and Banich, 2000; Weissman et al., 2000) It is also possible that the presence of a distractor served as a nonspecific endogenous cue that primed participants to be more attentive to the task, or that participants formed an expectation that a distractor would appear (as this was the case on two-thirds of the trials) and hesitated to respond on no distractor trials because they were awaiting its presentation. Discrepancies between the present work and that of Hughes and Rutherford (2013) could also stem from differences in stimulus properties. Although our stimuli were selected to be as similar as possible to those used by Hughes and Rutherford in terms of their mean valence and arousal, there were likely some cross-study differences in the precise test images selected, as we set out to systematically manipulate the presence/absence of IM cues whereas Hughes and Rutherford did not. Cross-study differences in the proportion of scenes that motivated approach or avoidance responses (which could impact laterality effects; e.g., see Balconi et al., 2017; Harmon-Jones and Gable, 2018), and/or in sample characteristics (e.g., sex distribution, alexithymic traits, and rates of SPS) may also have contributed to mixed findings. It is also possible, however, that this paradigm simply does not assess hemispheric asymmetries in scene perception reliably.

### Valence Processing

Overall, participants classified positive scenes more quickly and accurately than negative scenes. Processing positive scenes may normally be prioritized in the visual cortex, as activity here is elevated when static scenes with positive (vs. negative or neutral) valence are presented briefly (Schettino et al., 2019). Typical adults also show greater responsivity within the frontoparietal and lateral occipital cortices when viewing pleasant compared to unpleasant scenes matched for intensity (Frank and Sabatinelli, 2019). These findings are important, as superior processing of negative stimuli is often emphasized in the literature, given the obvious evolutionarily advantages it confers (Borgomaneri et al., 2014). It is possible that our participants were quick to *attend to* negative stimuli, but had more difficulty *classifying* these stimuli due to the larger number of response options that must be weighed when one is in an unpleasant situation (e.g., positive stimuli generally motivate approach, whereas negative stimuli can motivate approach, avoidance, or freezing responses; Kuhbandner et al., 2016; Roelofs, 2017). Incorporating eye-tracking in future studies could help to assess individual differences in how quickly attention is deployed to particular features in scenes.

Unlike participants scoring low in alexithymia, those with moderate-to-strong alexithymic traits found positive scenes as difficult to classify as negative scenes. This might be expected if they show a smaller “pleasure bias” in neural reactivity to positive scenes, and/or generally pay less attention to positively valenced scene content than lexithymic individuals. Koven (2014) suggested that the tendency for individuals with strong alexithymic traits to undervalue appetitive stimuli may reflect the fact that they resonate less with these stimuli at a physiological level, or that they struggle to make sense of the arousal that these stimuli generate (i.e., that they experience decoupling).

A possible consequence of undervaluing positive situations is that it may limit approach (exploration) when one is in a novel environment. Undervaluing positive situations could also contribute to the problems with cognitive reappraisal and emotion regulation that are frequently described in those with alexithymia (Walker et al., 2011). In this regard, it is important to comment on the fact that the MA and HA groups performed similarly on the emotional scenes task, even though the majority of individuals in the MA group actually scored in what would traditionally be called the “lexithymic” range on the TAS-20. This suggests that it may be important to control for sub-clinical alexithymic traits in future studies of emotion perception.

Why were *group differences* not apparent in judgments of negative scenes, overall? The answer to this question may lie in the fact that the stimuli selected in the present work had only moderate arousal ratings. Deng et al. (2013) found that adults with and without alexithymia showed different patterns of neural activity when viewing both low- and high-intensity positive scenes, but that group differences with negative scenes were only apparent with high-intensity stimuli. Future studies investigating the possibility of interactive effects of valence and arousal on scene perception in alexithymia are warranted.

### Processing of Emotional Scenes Depicting Implied Motion

IM increased the complexity of both positively and negatively valenced scenes, as evidenced by the fact that participants in the present study were generally slower and less accurate when classifying scenes that depicted IM – especially if they reported moderate-to-strong alexithymic traits. Viewing IM images normally produces stronger activity within the insula, medial temporal gyrus (Kolesar et al., 2017), fusiform gyrus (Michels et al., 2005), and superior temporal sulcus (Kourtzi and Kanwisher, 2000; Vaina et al., 2001; Kolesar et al., 2017) than viewing images that do not depict IM. In future work, it would be of interest to determine if the strength of these neural activations varies as a function of the strength of viewers’ alexithymic traits.

Some of the IM scenes used in the present study depicted human activities (e.g., someone skiing) or full-body/facial displays of emotion (e.g., an angry attack; a crying child). Participants may have been simulating these actions (Borgomaneri et al., 2012, 2014) *via* activation of the mirror neuron system (Calbi et al., 2017). This raises the possibility that atypicalities in motor simulation may have contributed to problems the MA and HA groups experienced when attempting to classify these scenes. Support for this idea comes from studies showing that those scoring high in alexithymia exhibit atypical facial motor responses to, and decreased mimicry of, facial expressions (Sonnby-Borgström, 2009; Scarpazza et al., 2015, 2017), and show heightened brain activation in the somatosensory cortex and supplementary motor area when viewing angry and fearful faces (Ihme et al., 2014).

Preliminary support for the idea that atypical integration of IM and emotion cues contribute to unusual patterns of embodiment in individuals with alexithymia comes from work by Borhani and colleagues. In an initial study, Borhani et al. (2015) reported that viewers typically showed slower RTs, lower accuracy, and heightened N190 amplitudes in both hemispheres when classifying images of bodies in action vs. at rest as either emotional or non-emotional, but that only the right hemisphere showed differential responding to fearful actions—implicating it in the integration of IM and emotion cues. They went on to show that exhibiting larger N190 amplitudes for emotional (particularly fearful) postures was characteristic of those reporting low but not high, levels of alexithymia (Borhani et al., 2016). From an evolutionary perspective, atypicalities in the integration of motion and emotion cues could put those with alexithymia at a disadvantage, for example, by negatively impacting how quickly or accurately they *evaluate* whether an approaching conspecific is friend or foe. This could explain why DDF (a marker of impaired emotional appraisal) was a negative predictor of accuracy in classification of scenes depicting IM.

In addition to scenes that included humans, some of the emotional stimuli used in the present study featured manipulable objects or scenes from nature. Martínez-Velázquez et al. (2017) reported that, whereas lexithymic individuals showed stronger electrodermal responses to scenes with (vs. without) social content, this effect was not seen in those reporting strong “affective” alexithymic traits (flattened affect and impoverished fantasy) and was reversed in those reporting strong “cognitive” alexithymic traits (that interfere with emotional appraisal). The way we embody aspects of non-social scenes can impact a range of non-social, perceptual and cognitive processes (Barsalou, 2008), such as the ability to estimate the size of goal-directed objects or judge the layout of the physical environment (Zadra and Clore, 2011). Future research should explore whether these kinds of processes are disrupted in individuals with alexithymia, particularly when the scenes are complex and depict IM.

### Externally Oriented Thinking

We found that EOT was a factor driving the relationship between alexithymia and task performance. Thus, individuals reporting stronger EOT classified fewer positive scenes as pleasant and were less likely to classify scenes with (vs. without) IM in a way that was consistent with normative scene valence ratings, compared to those reporting weaker EOT. The role of EOT makes sense if this particular trait is more strongly associated with disrupted embodiment than the other alexithymia dimensions, as suggested by Grynberg and Pollatos (2015).

Embodiment processes transform raw emotions into subjective feelings (Nummenmaa et al., 2018), which in turn, form the basis for ratings of pleasantness (Shiota et al., 2017). People scoring high on EOT may be hypo-reactive to emotional stimuli, and/or show diminished attention to their embodied (feeling) states. The latter suggestion is consistent with how Preece et al. (2017) characterize EOT in their attention-appraisal model of alexithymia. These authors contend that EOT and DIF/DDF influence distinct stages of the emotion regulation process; more specifically, they suggest that EOT impacts the *attention stage* by reducing focus on one’s emotional responses, whereas DIF and DDF (which limit the ability to understand one’s emotional experience) negatively impact the *appraisal stage*. By not directing attention inward, those scoring high in EOT may find it difficult to assess how environmental and body-based cues resonate internally and this, in turn, may make it harder for them to evaluate certain kinds of emotional scenes. This could create a situation where physiological and subjective reactions to a situation become decoupled (see Davydov et al., 2013).

### Alexithymia and Sensory Processing Sensitivity

We found that individuals scoring high on the HSPS were over-represented in the HA group. The strong positive correlations we observed between DIF/DDF scores and scores on the EOE subscale of the HSPS suggest that problems with emotional appraisal are most evident in those who are easily overwhelmed by busy sensory environments (see also Liss et al., 2008). The fact that both EOT and EOE were negative predictors of accuracy in the classification of positive scenes suggests that failing to direct attention inward (high EOT) or being hyper-reactive to sensory stimuli (high EOE) negatively impacts the evaluation of positive scenes. The fact that EOT and DDF were the only significant predictors of accuracy in the classification of IM scenes suggests that having a strong inward focus of attention may be particularly important for noticing the resonance generated by subtle motion cues, and that failing to attend to this resonance impacts appraisal of one’s affective response. One could speculate that EOE or LST might have accounted for more variance in performance had we used more complex dynamic stimuli, if the stronger activation of both sensory- and action-based neural pathways such stimuli would generate (Barsalou, 2008) was more likely to make those with sensory sensitivity uncomfortable. This is a question for future research.

The present results suggest that researchers interested in alexithymia should screen for SPS and vice versa, as a proportion of the population likely meet criteria for both traits and both are characterized by atypicalities in sensory processing. We would predict that individuals displaying *co-occurring* alexithymia and SPS would show relatively *weaker* EOT, and relatively *better* fantasizing abilities, than individuals who have alexithymia but not SPS, given that EOT and problems fantasizing are in many respects antithetical to some features of SPS. Indeed, individuals with SPS are typically characterized as introspective, “deep” processors with rich inner lives (Aron et al., 2012; Lionetti et al., 2018). The AES score of the HSPS (which was negatively correlated with EOT in the present sample) captures aspects of this cognitive style, albeit imperfectly. The possibility that different subtypes of alexithymia can be identified deserves more consideration, as doing so may help to explain discrepant results in the literature.

### Limitations and Future Directions

We chose to use IAPS images in the present work, in part, to keep our task as similar as possible to that used by Hughes and Rutherford (2013). Although the IM images that we selected appear to have captured some of the complexity and richness of natural scenes (as suggested by the finding that the presence of IM impacted task performance), future work that uses more ecologically valid stimuli (e.g., videos of emotional scenes; exposure to virtual reality or real-life situations) is warranted. Another limitation of the current work is that we did not include emotionally neutral scenes. It would be interesting to incorporate neutral stimuli into the study design to disentangle the effects of IM and emotion. In past work (e.g., Kolesar et al., 2017), functional overlap has been observed between neural regions that process IM and emotion. It may be that valenced IM scenes would elicit a different pattern of behavioral results than neutral IM scenes.

We used the TAS-20 to assess alexithymic traits. Bagby et al. (2020) provided a comprehensive review of this instrument. They state that, although it generally has good psychometric properties, in some studies the internal consistency of the EOT subscale has been found to be rather low, even though it correlates strongly with other constructs related to alexithymia. Given this, it will be important to replicate the current findings. Future researchers should consider supplementing the TAS-20 with an objective measure of alexithymia. Incorporating measures to assess the prevalence of past/present diagnoses of clinical depression, anxiety, or psychiatric disorder would also be useful. Although we failed to do this, we did rule out the possibility that negative affect in the moment accounted for our results.

Finally, we have reviewed literature suggesting that understanding one’s subjective *feelings* about a scene likely requires processing of how the scene “resonates” in sensory/sensorimotor networks, in the autonomic nervous system, and in parts of the emotional brain (see Shiota et al., 2017; Nummenmaa et al., 2018). But it is important to note that top-down factors also undoubtedly influence the extent to which we are aware of and are able to articulate how we feel in a given context (e.g., Gallese, 2014). It is quite possible that alexithymia reflects atypicalities in both bottom-up and top-down processes. It is also possible that subtypes of alexithymia may differ in the extent to which these two types of processes are compromised. Our focus has been on bottom-up processes; examining whether top-down factors mediate the relationship between specific facets of alexithymia and outcomes in different areas is an important avenue for future research.

## Conclusions

In line with previous recommendations (Borgomaneri et al., 2012), the current work highlights the importance of accounting for IM when investigating scene perception, or emotion processing more generally, using static images. We also found that individual differences in alexithymic traits (EOT, DDF) and sensory sensitivity (EOE) impacted task performance. Learning more about how individuals displaying specific traits, or alexithymia subtypes, respond to different environmental/contextual cues will enhance our understanding of the processing of emotional stimuli and advance alexithymia theory. Our findings may also have important clinical implications given that alexithymia is considered to be an important, transdiagnostic risk factor for a wide range of psychopathologies (Grynberg et al., 2012). Learning more about how alexithymia relates to the ability to evaluate and regulate one’s responses to stimuli in context may have important implications for understanding and treating those with behavioral addictions or mood/anxiety disorders, who often score high on alexithymia (Taylor et al., 2018).

## Data Availability Statement

The datasets presented in this study can be found in online repositories. The names of the repository/repositories and accession number(s) can be found below: https://doi.org/10.5203/FK2/MDLANS.

## Ethics Statement

The studies involving human participants were reviewed and approved by Psychology/Sociology Research Ethics Board, University of Manitoba. The patients/participants provided their written informed consent to participate in this study.

## Author Contributions

Authors are listed in order based on the importance of their contribution. SR was involved in all aspects of the research. LJ contributed to data analyses and writing the initial draft. PP and BS provided critiques and contributed to the final write-up. All authors contributed to the article and approved the submitted version.

### Conflict of Interest

The authors declare that the research was conducted in the absence of any commercial or financial relationships that could be construed as a potential conflict of interest.
